# Innovative application of new media in visual communication design and resistance to innovation

**DOI:** 10.3389/fpsyg.2022.940899

**Published:** 2022-08-03

**Authors:** Ge Yu, Shamim Akhter, Tribhuwan Kumar, Geovanny Genaro Reivan Ortiz, Kundharu Saddhono

**Affiliations:** ^1^College of Fine Arts, Shanxi University, Taiyuan, China; ^2^School of Languages, Civilization and Philosophy, Universiti Utara Malaysia, Changlun, Kedah, Malaysia; ^3^Prince Sattam Bin Abdulaziz University, Al-Kharj, Saudi Arabia; ^4^Catholic University of Cuenca, Cuenca, Ecuador; ^5^Indonesia Language Education Study Program, Universitas Sebelas Maret, Surakarta, Indonesia

**Keywords:** visual expression design with IT, flexible layout, diversified modes of transmission, interactivity of integration, resistance to innovative change, visual communication design

## Abstract

It has become essential to create and apply new media in visual communication design due to social media existence. This study aims to investigate the role of innovative applications of new media in visual communication design in educational institutions. Traditional media design in visual communication lacks to disseminate information more effectively, which requires innovative change. Therefore, this study attempts to highlight the role of innovative application of new media in visual communication by considering visual expression design with information technology (IT), flexible layout, diversified modes of transmission, and interactivity of integration. For this purpose, this study adopted a quantitative research approach in which a cross-sectional research design is followed. A questionnaire survey is carried out to collect data from educational institutions in China. Partial Least Square-Structural Equation Modeling (PLS-SEM) is used for data analysis. Results of the study indicated that innovative applications of new media have central importance in visual communication. However, resistance to innovative change has a negative role in the relationship between innovative applications of new media and visual communication design. Results of the study highlighted that visual expression design with IT, flexible layout, diversified modes of transmission, and interactivity of integration have a positive effect on visual communication design. Therefore, among the educational intuitions of China, implementing innovative applications related to the new media can lead to visual communication design. The results of this study provided several insights for the practitioners to promote communication methods among educational institutions.

## Introduction

Visual communication is the conveyance of ideas and information by using a broad range of forms that can be visually observed. These include different signs, diagrams, graphic designs, illustrations, animations, colors, and electronic resources (Kujur and Singh, 2020; Ji and Lin, 2022). Visual communication is a powerful communication tool that is central to communication (Ji and Lin, 2022; Xiangwei, 2022). It is based on animated Graphics Interchange Formats (GIFs), screenshots, different types of videos, pie charts, and infographics, along with slide deck presentations. All these types of visual communication have a vital role in the communication process through different methods. Along with other institutions, visual communication is standard in educational institutions as it provides different ways for teachers to teach effectively and lead to effective learning. Visual communication is an effective tool in educational institutions (Matusitz, 2005), but it is lacking in Chinese institutions.

Visual communication requires innovative applications (Goransson and Fagerholm, 2018; Liu, 2018). Mainly, new media applications in visual communication design are most important to introduce in Chinese educational institutions. Traditional communication design lacks different elements, which may lead to a lack of communication. The problems in traditional communication design can be well managed with the help of innovative applications of new media. New and traditional media complement each other and develop together (Yue, 2020). However, new media has the potential to create a better communication environment which can lead to the accuracy of communication. The innovative applications in new media can cover a broader range than traditional media. Advance technology in new media has several forms which can perform better than traditional media in different ways (Yue, 2020).

Earlier studies investigated visual communication through different aspects (Russmann, 2019; Duan, 2022). However, the element of innovation is rarely addressed. Different studies on visual communication design, such as Guan-Chen and Ko (2022), discussed the wireless data transmission technology about the Blockchain big data information presentation. Wang (2021) highlights visual communication design with the help of artificial intelligence and innovation development. Arsyadi (2019) discussed visual communication design in classrooms to facilitate instructors teaching at educational institutions. Huang and Zhou (2022) conducted a research study on visual communication design by considering the training program of universities. All these studies highlighted the essential findings and provided several implications.

However, the role of innovative applications of new media is not investigated. To get maximum benefits from visual communication, the role of innovative applications is required. Previously, research has investigated the impact of narrative theory on perspective development in graphic designing courses (Yang and Hsu, 2017). The study did not find the impact of the application of new media on visual communication design. Therefore, based on narrative theory, the current research examines the impact of the application of new media on visual communication design. This study attempts to highlight the role of the innovative application of new media in visual communication. Thus, to address the role of new media on visual communication, this study addressed four significant elements; visual expression design with information technology (IT), flexible layout, diversified modes of transmission, and interactivity of integration.

The innovative application of new media faces resistance to innovative ideas. The implementation of innovative ideas faces resistance to innovative change. The employees working in educational institutions resist innovative change. Previous studies also mentioned that resistance to innovation causes a decrease in the implementation of innovative ideas among institutions (Koch, 2021; Friedman and Ormiston, 2022; Naveed, 2022). Similarly, in visual communication design, resistance to innovative change is one of the significant problems. Hence, this study is one of the attempts to address the effect of resistance to innovative change in visual communication design. Finally, this study aims to investigate the role of innovative applications of new media in visual communication design in educational institutions. This study has vital theoretical contributions, which further lead to several practical implications for fostering visual communication design through the innovative application of new media.

## Literature review

### Visual expression design with information technology

IT (information technology) is the use of networking among storage, computers, and other physical infrastructures, devices, and processes that create, secure, store, exchange, and process all forms of electronic data. IT intended for financial gain encompasses both telecommunication and computer technology. As a past study conducted by Cebollada (2021), the visual form of data has a significant role in all forms of electronic data. It is because visual expressions are based on values, ideas, and feelings that are visually presented. In addition, Erickson (2020) described that visual expression design is something perceptible that is perceived on a screen.

According to the present study, visual expression design with IT refers to acceptable, tolerable, standard, and decent designs in every aspect of IT, mainly to depict the actual meaning of the cause behind the creation of the design. Raposo (2018) says that visual communication is an art that combines words, thoughts, images, and ideas to express a message or to convey information aiming to produce a particular effect. Visual expression design with IT is more effective, persuasive, convincing, and result-oriented because IT adds significant meaning to the growth of the business and commerce sector by generating the maximum possible outcome.

#### Flexible layout

To create consistency, avoidance of unnecessary items is necessary. However, a container must be able to hold all the items that are specified and unspecified. Sumual (2021) represented that a potent layout is always invisible to users and provides more comfort to get things done more quickly. A user-friendly and flexible layout is one of the primary purposes of any developer. A study on creating a dynamic layout for a virtual environment for walking conducted by Vasylevska (2013) says that a dynamically practical layout is called a flexible layout. It provides adequate methods to distribute, align, and space among items in a container or stack even when the size of the items is dynamic or unknown. Hence, a flexible layout is an art to fit the requirements of a system. Phoon (2018) presented that a flexible layout helps expand items to cover free space or shrinks the items to avoid overflow.

Moreover, Pourvaziri et al. (2021) described that regular layouts are limited which are vertically or horizontally based. While the flexible layout is direction-agnostic, it allows the automatic position of items by wrapping. In addition, Haddou and Benyoucef (2019) described that a flexible layout is always ready to accept products of uncertain sizes. Therefore, a flexible layout is crucial for accomplishing dynamic manufacturing cells, particularly in an environment with dynamic production.

#### Diversified modes of transmission

**FIGURE 1:**
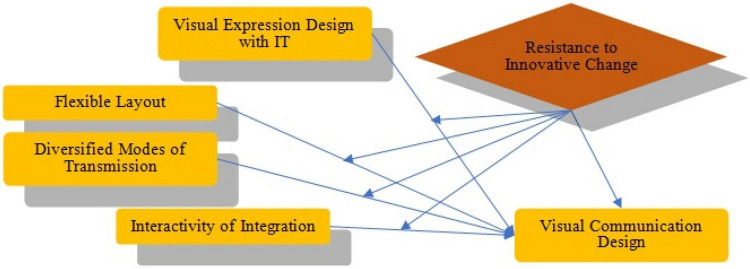
Research model.

**FIGURE 2:**
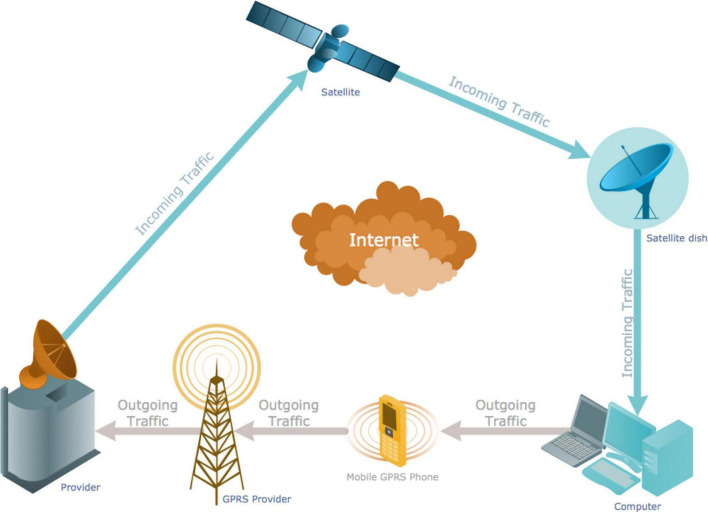
Telecommunication network. Source: https://www.conceptdraw.com/How-To-Guide/picture/Computer-and-Networks-Telecommunication-Network-Diagrams-GPRS-network-scheme.png. VED-IT, Visual Expression Design with IT; FL, Flexible Layout; DMT, Diversified Modes of Transmission; II, Interactivity of Integration; VCD, Visual Communication Design; RI, Resistance to Innovative Change.

In telecommunication, transmission refers to the spreading or broadcasting of information. A transmission system sends a signal from place A to place B. Figure 1 shows a telecommunication network representing the current transmission mode. Figure 2 show the complete networking of telecommunication.

The transmission system sends information consisting of video or audio or some other type of data, in digital or analog form using optical or electromagnetic signals between various sites. Bezruk et al. (2012) researched multi-criteria optimization in telecommunication network planning, designing, and controlling. They described the following four types of telecommunication networks: (a) public-switched telephone networks, (b) radio networks, (c) packet-switched networks, and (d) computer networks.

The traditional modes of transmission are relatively simple. However, in computers, transmission modes are comparatively complex and more efficient (Cheng, 2019). Furthermore, according to Liu (2017), in computers, simple mode, half-duplex mode, and full-duplex mode are the three primary modes of transmission. Gao (2015) also determined that communication in simple mode is unidirectional, which means that between two devices, one will transmit while the other will receive. Lari and Gandomani (2022) explored that in a half-duplex mode of transmission, both the devices can transmit and receive data but not simultaneously. While in full-duplex, both the devices can simultaneously transmit and receive data.

#### Interactivity of integration

Innovation in visual communication design is necessary to use modern technologies because the innovative designs of products have become hot items produced by professional designers. Moreover, Chen and Zheng (2021) concluded that visual communication design’s professionally designed items are integrated with modern technologies. A previous study by Yue (2020) explored that interactivity of integration in visual communication design helps bring unique information that can be linked to design requiring more innovative creations. The interactivity of integration also refers to the process of designing in which a designer can display his/her work in a customized way; hence, viewers’ attention is increased to see more exciting content generated by the designer. In addition, Kim and Lee (2019) proved that interactivity of integration attracts viewers to participate in the designing process. Sometimes using advanced technologies that help achieve a higher sense of integration and incorporation aims to achieve higher quality and ensure that more decadent designs promote audiences.

#### Resistance to innovative change

Like any change, innovation is also a change that requires intention, effort, and motive; it needs leadership and vision. Furthermore, it generates resistance. In several cases, an increase is the primary goal, meaning innovation is mandatory in these cases. Kostis (2018) described that innovative changes have consequences that influence the performance in either positive or negative ways. According to Zhou et al. (2019), resistance to innovative change refers to the limitation that decreases or stops increasing the performance volume. In other words, resistance to innovation change is a factor that spectacle the progressive boundaries of an institution. King (2007) determined that resistance to innovative change decreases the demanding work contexts that ultimately result in organizational outcomes. In addition, resistance to innovation change is defined as the act toward the acceptance and usage of any modernization that results in continuing the current situation and resisting any abnormality from prevailing beliefs. However, resistance to innovative change is categorized based on various factors such as rational, logical, emotional, psychological, and sociological resistance. According to Talwar (2020), lack of trust, poor communication, emotional response, fear of failure, surprises, constant change, and listening to employees are the common reasons for resistance to innovative change.

#### Visual communication design

Scholars have long been concerned about the design principles such as unity, proportion, complexity, and symmetry (Wang and Hsu, 2020; Yanarates and Zhou, 2021; Haapala, 2022). According to Liang (2021), visual communication design is a process that combines technology and visual arts to communicate thoughts and creative ideas. Typically, every visual communication design begins with a message transformed into visual communication that goes beyond complete pictures and words. Zhang and Wu (2021) presented that broadcast, film, advertising, web, publishing, television, and industry are the visual channels and formats for creative visual material. Modern means of communication such as social media platforms and websites keep bringing current visual communications design to attract their audience.

Furthermore, typography and art also significantly positively affect visual communication design. Hence the fundamental purpose of visual communication design is to convey information. However, there is a difference between graphic design and visual communication design. According to Emans and Murdoch-Kitt (2018), every image that people see is graphic design. Only the images that convey a message are visual communication design, while the images that do not convey a message are graphic design.

#### Development of hypotheses

##### Visual expression design with information technology and visual communication design

A study by Jia (2017) explored that visual expression design plays a significant role in educational institutions. Educational institutions, especially in China, without visual expression design, fail to meet their targets, particularly concerning learning and teaching methods. In addition, Saris (2020) determined that learning becomes more compelling and persuasive for students through clear, attractive, engaging, and appropriate visual expression design. Several previous studies are also evident that increasing the value of visual expression design, especially in educational institutions, increases the value of the institutions (Wu, 2019; Huang, 2021). Furthermore, visual expression design with IT adds significant meaning to the visual communication design. Prior literature also revealed that visual expression design with IT adds significant meaning to educational materials such as videos, infographics, animated GIFs, and slide presentations that make the massage or information clear, concise, and succinct; hence, learners easily understand. Previous literature shows that an increase in the value of visual expression design with IT also increases the value of visual communication design. Hence, it is enclosed.

***H1:*** *Visual expression design with IT positively correlates with visual communication design.*

##### Flexible layout and visual communication design

A flexible layout helps to design a visual communication design. According to the present study, the flexible layout has a significant favorable influence on the teaching methods, especially for teachers in China. It is observed that innovation in teaching and education increases the performance of the teachers and the educational institutions. Furthermore, Huang L. (2020) determined that during the COVID-19 outbreak, the flexible learning process was entirely ensuring for quality of education and increased the value of visual communication design by adding significant positive meaning to the online learning process. Ozkan (2021) explored that the faculty of an educational institution feel more comfortable with movement in the flexibility and engagement in technology-enhanced classrooms. In addition, Zahlbruckner (2021) determined that flexible layout significantly affects visual communication design. Results of the previous study show that increasing the value of flexible layouts also increases the value of visual communication design. Hence, it is enclosed.

***H2:*** *Flexible layout has a positive relationship with visual communication design.*

##### Diversified modes of transmission and visual communication design

For higher education institutions, diversified modes of transmission play a crucial role (Flückiger, 2021). In countries like China, educational institutions are responsible for students’ behavior, personality building, career orientation, and socio-economic values. Therefore, diversified modes of transmission in educational institutions in the country are more significant. Previous literature shows that educational institutions in China having good value for diversified modes of transmission remain successful in building visual communication designs that are advantageous both for the teaching methods and learning process (Talidong and Toquero, 2021). Furthermore, Wu (2021) determined that diversified modes of transmission have positive effects on the health of teachers and students. During the COVID-19 outbreak, the value for visual communication design increased due to the variety of diversified modes of transmission. Hence, it is clear from the results of the previous study that increasing the value of diversified modes of transmission also increases the value of visual communication design. Therefore, it is enclosed that:

***H3:*** *Diversified modes of transmission have a positive relationship with visual communication design.*

##### The interactivity of integration and visual communication design

Yadav and Prakash (2022) determined that interactivity of integration plays a significant role, especially in the sustainable development of educational institutions. Furthermore, interactivity of integration helps assess the management of educational institutions in countries like China and India. Turnbull (2021) also agreed that interactivity of integration helps to deal with the challenges such as communication gaps. The present study concluded from the previous literature that several kinds of barriers, such as physical, emotional, cultural, perceptual, language, and interpersonal barriers, are the real reasons that ultimately lead to communication gaps. However, decreased value of interactivity of integration is the real cause behind these barriers (Vakulenko, 2021). Studies also determined that the value of the visual communication design increases with the increase in the interactivity of integration. Furthermore, the educational institutions in China have better versions of visual communication design that increased value for interactivity of integration. Therefore, it is enclosed that:

***H4:*** *Interactivity of integration has a positive relationship with visual communication design.*

##### Resistance to innovative change and visual communication design

Kujur and Singh (2020) investigated that effective visual communication design, especially in an educational institution, particularly in a country like China, helps students and learners to understand their lessons and other information. Faculty often need visual communication designs that can convey their message correctly to their pupils. Moreover, the faculty also demands innovation in visual communication design. However, Frost (2019) determined that resistance to innovative change in the learning process and teaching methods eventually results in a communication gap. In addition, Demuyakor (2020) also explored that during the COVID-19 outbreak, the learning process in higher institutions of education in China was diminished. It was due to resistance to innovative change because of COVID-19. Outcomes of the previous literature result in a decrease in the value of resistance to innovative change and a decrease in the value of visual communication design. Hence, it is enclosed:

***H5:*** *Resistance to innovative change has a negative relationship with visual communication design.*

#### Moderation relationship

Educational institutions in China having increased value of resistance to innovative change have adverse effects on the relationship between visual expression design with IT and visual communication design. Huang W. (2020) determined that resistance to innovative change results in reduced performance in educational institutions. Moreover, the moderation role of resistance to innovative change decreases the value of the visual expression design with IT and visual communication design. Xie (2022) concluded that resistance to innovative change decreases the value of talent in learners; hence, the value of intelligent computing decreases. Hence, it is enclosed:

***H6:*** *Resistance to innovative change moderates the relationship between visual expression design with IT and visual communication design*

Resistance to innovative change in the educational institutions in China results in adverse effects on the relationship between flexible layout and visual communication design. Liu (2020) investigated that a flexible classroom layout pattern adds significant meaning to students’ learning process in educational institutions. Moreover, the moderating role of resistance to innovative change decreases the value of flexible layout, eventually decreasing the value of visual communication design. Prior literature on the educational system also shows that resistance to innovative change decreases the value of educational institutions (Yan, 2012). Hence, it is enclosed:

***H7:*** *Resistance to innovative change moderates the relationship between flexible layout and visual communication design*

Educational institutions in China having increased value of resistance to innovative change have adverse effects on the relationship between diversified modes of transmission and visual communication design. According to Kaur (2020), resistance to innovative change always creates boundaries between the target and the current performance of an educational institution. Furthermore, the moderating role of resistance to innovative change decreases the value of diversified modes of transmission, ultimately resulting in a decrease in the value of visual communication design. Results of the previous study show that resistance to innovative change has sufficient influence on diversified modes of transmission and visual communication design (Haotian, 2020). Hence, it is enclosed:

***H8:*** *Resistance to innovative change moderates the relationship between diversified modes of transmission and visual communication design*

In China, educational institutions that carry resistance to innovative change show adverse effects on the relationship between interactivity of integration and visual communication design. Shahbaz (2019) determined that needless interference with selective perception, loss of freedom, security, threats to the power of influence, skill obsolescence, need fulfillment, habit, and economic implications are the reasons behind resistance to innovative change. In addition, Blin and Munro (2008) explored that the moderation role of the resistance to innovative change decreases the value of interactivity of integration, ultimately resulting in a decrease in the value of visual communication design. Hence, it is enclosed:

***H9:*** *Resistance to innovative change moderates the relationship between interactivity of integration and visual communication design*

## Research methodology

The current study examined the relationship among visual expression design with IT, flexible layout, diversified modes of transmission, interactivity of integration, visual communication design, and resistance to innovative change. The nature of this relationship is supported by a cross-sectional research design. Therefore, the current study followed a cross-sectional research design by following the quantitative research approach. While using a cross-sectional research design, this study carried out a questionnaire survey to collect data from respondents.

The population of the study is based on the educational institutions in China. The teachers and employees working in these institutions were considered the study’s respondents. Few previous studies investigated visual expression design with IT, flexible layout, diversified modes of transmission, interactivity of integration, visual communication design, and resistance to innovative change. However, these elements are rarely discussed among Chinese educational institutions. Therefore, this study conducted a questionnaire survey to collect data from Chinese educational institutions. The questionnaires were distributed by using simple random sampling among the educational institutions. This study distributed 500 questionnaires and 225 questionnaires were returned; 10 questionnaires were missing by a significant part, therefore, excluded from the survey; and 215 valid responses were used in the data analysis.

### Measures

Visual communication design is measured by considering the effectiveness in educational institutions. Five items are used to measure visual communication design. Visual expression design with IT is measured by using the role of IT in visual expression design. Flexible layout is measured by using the different layouts and their effectiveness in visual communication among the educational institutions. Visual expression design with IT and flexible layout are measured using five items each.

Furthermore, four items are used to measure diversified modes of transmission, and these items are designed using different transmission modes. The interactivity of integration is also measured using four scale items based on the interactivity, which are regarding time and technology. Finally, four items are used to measure resistance to innovative change. These items are developed by considering the resistance to innovative change by the employees. After the development of the questionnaire, face validity and content validity are confirmed by reviewing the questionnaire from experts in a related field. After that, a pilot study is carried out by using 100 questionnaires. Additionally, exploratory factor analysis (EFA) is also employed.

### Data analysis

The current study analyzed data using Partial Least Square-Structural Equation Modeling (PLS-SEM). PLS-SEM is the most well-known data analysis technique recommended by several previous studies (Hair, 2020; Hair et al., 2021). PLS-SEM is based on two significant steps: (1) measurement model assessment and (2) structural model assessment. However, before data analysis, the current study performed initial data screening in which missing values and outliers are examined. After data screening, the data statistics are given in Table 1.

**TABLE 1:** Data statistics.

	No.	Missing	Mean	Median	Min	Max	SD	Kurtosis	Skewness
VED-IT1	1	0	3.488	3	1	7	1.849	–0.675	0.32
VED-IT2	2	0	3.668	3	1	7	1.796	–0.634	0.298
VED-IT3	3	0	3.093	3	1	7	1.503	–0.147	0.613
VED-IT4	4	0	3.229	3	1	7	1.524	0.34	0.881
VED-IT5	5	0	3.244	3	1	7	1.485	0.63	0.899
FL1	6	0	3.185	3	1	7	1.48	0.369	0.769
FL2	7	0	3.137	3	1	7	1.428	0.347	0.668
FL3	8	0	3.249	3	1	7	1.54	0.169	0.682
FL4	9	0	3.166	3	1	7	1.489	0.435	0.838
FL5	10	0	3.049	3	1	7	1.413	–0.11	0.477
DMT1	11	0	3.215	3	1	7	1.401	0.306	0.641
DMT2	12	0	3.141	3	1	7	1.463	0.124	0.647
DMT3	13	0	3.122	3	1	7	1.527	0.049	0.687
DMT4	14	0	3.259	3	1	7	1.427	–0.202	0.521
II1	15	0	3.19	3	1	7	1.371	0.247	0.59
II2	16	0	3.059	3	1	7	1.364	–0.221	0.533
II3	17	0	3.18	3	1	7	1.333	0.208	0.611
II4	18	0	3.18	3	1	7	1.439	–0.115	0.68
RI1	19	0	3.215	3	1	7	1.453	0.019	0.66
RI2	20	0	3.234	3	1	7	1.359	0.185	0.683
RI3	21	0	3.356	4	1	7	1.606	–0.593	0.152
RI4	22	0	3.298	4	1	7	1.751	–0.74	0.289
VCD1	23	0	3.361	4	1	7	1.957	–0.916	0.362
VCD2	24	0	3.346	3	1	7	2.032	–1.067	0.393
VCD3	25	0	3.215	3	1	7	2.147	–1.075	0.506
VCD4	26	0	3.278	3	1	7	2.014	–0.972	0.463
VCD5	27	0	3.366	3	1	7	1.722	–0.63	0.374
VCD6	28	0	3.332	3	1	7	1.955	–0.917	0.379

VED-IT, Visual Expression Design with IT; FL, Flexible Layout; DMT, Diversified Modes of Transmission; II, Interactivity of Integration; VCD, Visual Communication Design; RI, Resistance to Innovative Change.

### Measurement model assessment

**FIGURE 3:**
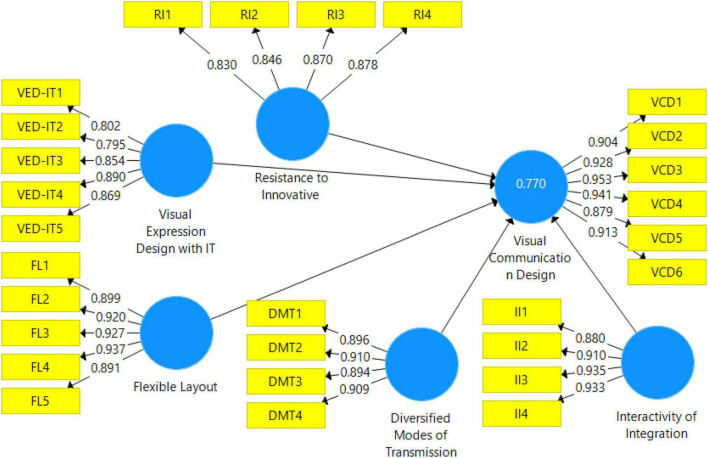
Measurement model. VED-IT, Visual Expression Design with IT; FL, Flexible Layout; DMT, Diversified Modes of Transmission; II, Interactivity of Integration; VCD, Visual Communication Design; RI, Resistance to Innovative Change.

The PLS measurement model is highlighted in Figure 3. In this step of data analysis, reliability and validity are considered. While considering the reliability, this study preferred to examine factor loadings and composite reliability (CR). According to the literature, factor loadings must not be less than 0.7 (Hameed, 2020, 2021; Hair et al., 2021). Results shown in Table 2 highlighted that all the scale items have factor loading higher than 0.7.

**TABLE 2:** Factor loadings, reliability, and convergent validity.

Variables	Items	Loadings	Alpha	CR	AVE
Diversified Modes of Transmission	DMT1	0.896	0.924	0.946	0.814
	DMT2	0.91			
	DMT3	0.894			
	DMT4	0.909			
Flexible Layout	FL1	0.899	0.951	0.963	0.838
	FL2	0.92			
	FL3	0.927			
	FL4	0.937			
	FL5	0.891			
Interactivity of Integration	II1	0.88	0.935	0.953	0.837
	II2	0.91			
	II3	0.935			
	II4	0.933			
Resistance to Innovative	RI1	0.83	0.88	0.916	0.733
	RI2	0.846			
	RI3	0.87			
	RI4	0.878			
Visual Communication Design	VCD1	0.904	0.964	0.971	0.846
	VCD2	0.928			
	VCD3	0.953			
	VCD4	0.941			
	VCD5	0.879			
	VCD6	0.913			
Visual Expression Design with IT	VED-IT1	0.802	0.898	0.924	0.71
	VED-IT2	0.795			
	VED-IT3	0.854			
	VED-IT4	0.89			
	VED-IT5	0.869			

VED-IT, Visual Expression Design with IT; FL, Flexible Layout; DMT, Diversified Modes of Transmission; II, Interactivity of Integration; VCD, Visual Communication Design; RI, Resistance to Innovative Change.

Furthermore, composite reliability (CR) is reported in Table 2. It must be higher than 0.7 for all variables. This study achieved the minimum level of CR, as shown in Table 2. Additionally, to check the convergent validity, this study preferred the average variance extracted (AVE), which must be higher than 0.5. AVE higher than 0.5 confirmed the convergent validity, as shown in Table 2.

Finally, in the measurement model, this study considered discriminant validity, which is essential before examining the relationship between variables. Two methods are used to confirm the discriminant validity. First, the heterotrait-monotrait ratio of correlations (HTMT)_0.9_ is considered because all the values must not be higher than 0.9. HTMT is highlighted in Table 3. AVE square root is considered, which is highlighted in Table 4.

**TABLE 3:** Discriminant validity (HTMT).

	Diversified Modes of Transmission	Flexible Layout	Interactivity of Integration	Resistance to Innovative	Visual Communication Design	Visual Expression Design with IT
Diversified Modes of Transmission						
Flexible Layout	0.825					
Interactivity of Integration	0.769	0.756				
Resistance to Innovative	0.782	0.653	0.64			
Visual Communication Design	0.73	0.71	0.662	0.821		
Visual Expression Design with IT	0.832	0.67	0.761	0.753	0.729	

**TABLE 4:** Discriminant validity (AVE Square Root).

	Diversified Modes of Transmission	Flexible Layout	Interactivity of Integration	Resistance to Innovative	Visual Communication Design	Visual Expression Design with IT
Diversified Modes of Transmission	0.902					
Flexible Layout	0.895	0.915				
Interactivity of Integration	0.801	0.901	0.915			
Resistance to Innovative	0.872	0.857	0.834	0.896		
Visual Communication Design	0.689	0.68	0.63	0.761	0.92	
Visual Expression Design with IT	0.856	0.9	0.888	0.838	0.68	0.843

### Structural model assessment

**FIGURE 4:**
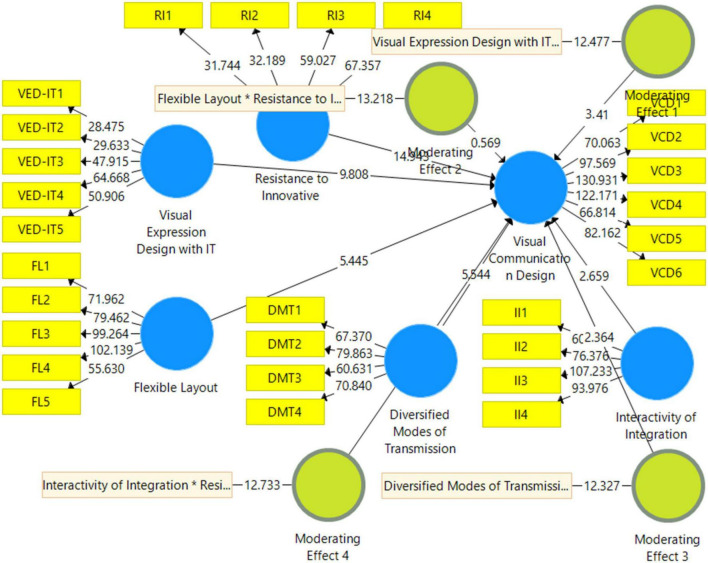
Structural model.

The structural model examines the relationship between visual expression design with IT, flexible layout, diversified modes of transmission, interactivity of integration, visual communication design, and resistance to innovative change. Figure 4 shows the structural model assessment, and the results are reported in Table 5. This study considered a t-value of 1.96 to accept the hypotheses. Results show that visual expression design with IT positively affects visual communication design with a t-value of 9.808. A flexible layout positively affects visual communication design with a t-value of 5.445.

**TABLE 5:** Results.

	Original Sample (O)	Sample Mean (M)	Standard Deviation (STDEV)	T Statistics (| O/STDEV|)	*P* values
Diversified Modes of Transmission - > Visual Communication Design	0.055	0.072	0.01	5.544	0
Flexible Layout - > Visual Communication Design	0.05	0.033	0.01	5.445	0
Interactivity of Integration - > Visual Communication Design	0.255	0.25	0.096	2.659	0.008
Moderating Effect 1 - > Visual Communication Design	−0.045	−0.034	0.013	3.41	0.001
Moderating Effect 2 - > Visual Communication Design	0.037	0.033	0.065	0.569	0.57
Moderating Effect 3 - > Visual Communication Design	−0.181	−0.182	0.077	2.364	0.018
Moderating Effect 4 - > Visual Communication Design	0.141	0.139	0.099	1.428	0.154
Resistance to Innovative - > Visual Communication Design	−0.89	−0.87	0.059	14.943	0
Visual Expression Design with IT - > Visual Communication Design	0.079	0.073	0.01	9.808	0

Furthermore, diversified transmission modes also positively affect visual communication design with a t-value of 5.544. Similarly, interactivity of integration positively affects visual communication design with a t-value of 2.659. However, resistance to innovative change harms visual communication design, with a t-value of 14.943.

**FIGURE 5:**
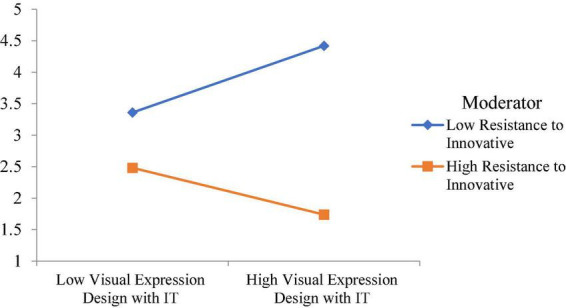
Moderating effect of resistance to innovative change weakens the relationship between visual expression design with IT and visual communication design.

**FIGURE 6:**
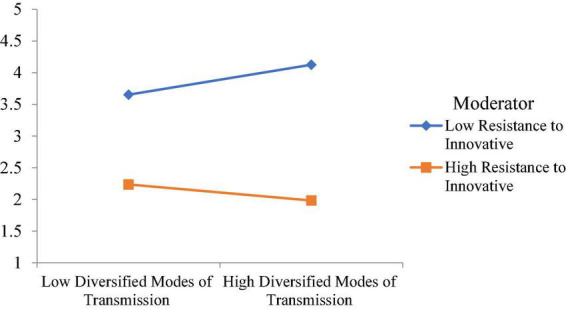
Moderating effect of resistance to innovative change weakens the relationship between diversified modes of transmission and visual communication design.

Moreover, this study examined the moderating role of resistance to innovative change. The moderating role of resistance to innovative change between visual expression design with IT and visual communication design is significant, with a t-value of 3.41. However, the moderating role of resistance to innovative change between flexible layout and visual communication design is insignificant. The moderating role of resistance to innovative change between diversified modes of transmission and visual communication design is significant, with a t-value of 2.364. Finally, the moderating role of resistance to innovative change between interactivity of integration and visual communication design is insignificant. The moderating effect of resistance to innovative change weakens the relationship between visual expression design with IT and visual communication design, as shown in Figure 5. Additionally, the moderating effect of resistance to innovative change weakens the relationship between diversified modes of transmission and visual communication design, as shown in Figure 6.

## Discussion and conclusion

The current study examined the relationship between visual expression design with IT, flexible layout, diversified modes of transmission, interactivity of integration, visual communication design, and resistance to innovative change. Nine hypotheses are proposed, including the five direct and four moderating effect hypotheses. After collecting data by using a questionnaire survey, PLS-SEM is employed to examine the relationship between variables.

Hypothesis 1 highlighted the significant and positive relationship between visual expression design with IT and visual communication design. It shows that introducing visual expression design with IT among the educational intuitions can improve the visual communication design. In line with these results, previous studies also highlighted that IT is essential in visual communication design (Andriyan and Anesti, 2020; Wu and Li, 2020; Guan and Wang, 2022). Hypothesis 2 considered the relationship between flexible layout and visual communication design. A positive and significant relationship was found between flexible layout and visual communication design. A better flexible layout has the potential to promote visual communication design.

Along with the current study, Yue (2020) highlighted the central role of layout in visual communication. According to Yue (2020), innovation in layout can promote visual communication design. Hypothesis 3 investigated the relationship between diversified modes of transmission and visual communication design. Similar to other hypotheses, this hypothesis is significant and positive, showing that diversified transmission modes have a positive role in influencing visual communication in educational institutions in China. Hypothesis 4 is also significant and positive, highlighting the positive role of interactivity of integration in visual communication. Thus, the adoption of innovative applications related to the interactivity of integration and diversified modes of transmission in visual communication design is influential among institutions, also supported by Yue (2020). Finally, while examining direct effect, hypothesis 5 indicated a relationship between resistance to innovative change and visual communication design. According to the results, resistance by individuals toward adopting innovative change has a negative role in visual communication.

Furthermore, the current study proposed five hypotheses regarding the moderating role of resistance to innovative change. Hypothesis 6 shows the moderating role of resistance to innovative change between visual expression design with IT and visual communication design. The moderating effect of resistance to innovative change weakens the relationship between visual expression design with IT and visual communication design. It shows that the increase in the level of resistance by the employees, teachers, and students in the educational institutions of China can lead to a decrease in the effect of innovative application of new media. Hypothesis 7 is not supported. However, hypothesis 8 shows the moderating role of resistance to innovative change between diversified modes of transmission and visual communication design. Results show that moderating effect of resistance to innovative change weakens the relationship between diversified modes of transmission and visual communication design. Therefore, the innovative application of new media cannot lead to visual communication in the presence of resistance to innovative change. Hypothesis 9 is not supported. Finally, the study results show that innovative applications of new media such as visual expression design with IT, flexible layout, diversified modes of transmission, interactivity of integration, and visual communication design can expedite visual communication among Chinese educational institutions. However, resistance from the employees, teachers, and students to adopt innovative change may decrease the effectiveness of new media applications.

### Theoretical implications

The current study addressed the vital part of the literature gap by considering the role of innovative applications of new media in visual communication design. Therefore, this study has important theoretical implications. First, this study addressed the visual communication design of the new media rather than traditional media, which is not addressed by earlier studies. Second, this study focused on the innovative applications of media, which are rarely studied in the literature. Third, although few studies addressed visual expression design with IT, flexible layout, diversified modes of transmission, and interactivity of integration, earlier studies have not considered these elements of the educational institutions. Mainly, the educational institutions of China are ignored by the previous studies. Four, several studies investigated visual communication design through different dimensions. However, previous studies have not employed PLS-SEM. Therefore, this study contributed methodologically to the field of visual communication design by using a new data analysis tool. Five, this study introduced resistance to innovative change as moderating variable, which is not considered by previous studies.

### Practical implications

The results of this study provided several insights for the practitioners to promote communication methods among educational institutions. This study highlighted the ignored part of the literature, therefore, this study has vital practical implications. First, as traditional media in visual communication design is lacking in performance, this study proposed innovative applications of new media that can foster visual communication performance. Second, the study results are helpful for the educational institutions of China to enhance communication methods which may strengthen the relationship between teachers and students, leading to better learning. Third, the study outcomes provided several insights for the practitioners to enhance visual communication by focusing on visual expression design with IT, flexible layout, diversified modes of transmission, and interactivity of integration. Four, the management of educational institutions must reduce the resistance to adopting innovation to foster visual communication among educational institutions in China. Educational institutions’ employees, teachers, and students should adopt innovative visual communication changes.

### Limitations and recommendations

The study’s shortcomings are due to the small sample size, which may make it difficult to generalize the results. Future research may thus evaluate the findings using a bigger sample size. Employees and educators from Chinese educational institutions were included in the research. In comparison to the 500 surveys issued, only 225 were returned. It demonstrates that respondents had little interest in taking part in the study. Therefore, metrics that can entice respondents to participate in the study can be included in subsequent research. Additionally, this study might be conducted among staff members at institutions of higher learning that do not provide instruction. As predictive factors for visual communication design, flexible layout, transmission methods, and interactivity of integration have been employed. To get more understanding findings on the aspects that might influence the visual communication design, this model can be modified with additional variables. Future research might also examine how visual communication designs relate to mobile APP interfaces. Future research might also look at how user behavior and technology adoption play a mediating effect. A language barrier may be a moderator in a future study on visual communication designs.

## Data availability statement

The original contributions presented in this study are included in the article/supplementary material, further inquiries can be directed to the corresponding author/s.

## Author contributions

GY conceived the concept. SA designed the concept. GO collected the data. TK wrote the manuscript. KS read and agreed to the published version of the manuscript. All authors contributed to the article and approved the submitted version.
